# Change in Excitability of Cortical Projection After Modified Catheter Balloon Dilatation Therapy in Brainstem Stroke Patients with Dysphagia: A Prospective Controlled Study

**DOI:** 10.1007/s00455-017-9810-6

**Published:** 2017-05-26

**Authors:** Xiaomei Wei, Fan Yu, Meng Dai, Chunqing Xie, Guifang Wan, Yujue Wang, Zulin Dou

**Affiliations:** 1 Department of Rehabilitation Medicine, The Third Affiliated Hospital, Sun Yat-sen University, No. 600, Tianhe Road, Guangzhou, 510630 Guangdong China; 2 Department of Rehabilitation Medicine, Shanghai General Hospital, No. 100 Haining Road, Hongkou District, Shanghai, 200080 China

**Keywords:** Brainstem stroke, Deglutition, Deglutition disorders, Balloon dilatation therapy, Corticobulbar, Motor evoked potential

## Abstract

Although the modified balloon dilatation therapy has been demonstrated to improve pharyngeal swallowing function post stroke, the underlying neural mechanisms of improvement are unknown. Our aims are (1) to investigate the effect of modified balloon dilatation on the excitability of corticobulbar projections to the submental muscle in dysphagic patients with brainstem stroke and (2) the relation between changes in excitability and pharyngeal kinematic modifications. Thirty patients with upper esophageal sphincter (UES) dysfunction due to unilateral brainstem stroke were recruited into two groups. The patients in dilatation group received modified balloon dilatation and conventional therapies, and those in control were only treated by conventional therapies (twice per day). The amplitudes of bilateral submental motor evoked potentials (MEPs) induced by transcranial magnetic stimulations over bilateral motor cortex, diameters of UES opening (UOD) and maximal displacement of hyoid (HD) were all assessed at baseline and the endpoint of treatments. Repeated ANOVA analysis revealed significant main effect of group, time and MEP laterality on MEP amplitudes (*p* = 0.02). There were no differences in the pretreatment measures between groups (all *p* > 0.05). After treatment, the amplitudes of affected submental MEP evoked by ipsilateral cortical pulse as well as UOD and HD were significantly different in dilatation group compared to control (amplitude: *p* = 0.02, UOD: *p* < 0.001, HD: *p* = 0.03). The differences of pre- and post-treatment amplitudes of the affected MEP evoked by ipsilateral stimulation showed a positive correlation with the improvement of HD (dilatation: *R* ^2^ = 0.51, *p* = 0.03; control: *R* ^2^ = 0.39, *p* = 0.01), rather than UOD in both groups (all *p* > 0.05). In conclusion, modified balloon dilatation therapy can increase the excitability of affected projection in patients with unilateral brainstem stroke.

**Electronic supplementary material:**

The online version of this article (doi:10.1007/s00455-017-9810-6) contains supplementary material, which is available to authorized users.

Swallowing is a complex task that requires the sensorimotor network of brainstem and widespread cortical regions [1, 2]. Up to 80% of patients with a brainstem injury have severe dysphagia [3], manifesting as coughing, choking, aspiration, penetration, food residue, and regurgitation, with some complications, including pneumonia, malnutrition, and dehydration. Failure of upper esophageal sphincter (UES) relaxation is a common finding of dysphagia for patients with brainstem injury [4], which increases the risk for aspiration because of bolus overflow into the airway.

Balloon dilatation therapy has been extensively used in the treatment of primary cricopharyngeal disorders and gastrointestinal tract strictures in recent decades [5–7]. The inflated balloon was used to passively stretch sphincter for several seconds without swallowing. The effectiveness of balloon dilation therapy in patients with neurological disorders, however, may vary depending on the cause of UES dysfunction. For example, while passive dilation may decrease UES pressure in persons with cricopharyngeal achalasia [8–10], it may be of little benefit to patients with brainstem strokes with a decrease in UES resting pressure. In a previous study, we examined the efficacy of a modified balloon dilatation where patients were asked to conduct a voluntary task-oriented swallowing guided by a balloon with graded volumes. The balloon will move through UES due to the opening of UES during voluntary swallowing instead of passive dilation, which was proved to be more effective than the passive approach [11]. This approach generated long-term improvements in swallowing function, as indicated by increase in functional oral intake scale (FOIS) scores, and in UES opening, and excursions of the hyoid bone as measured by videofluoroscopy [12]. Furthermore, the efficacy in increasing UES relaxation time, strengthening pharyngeal propulsion, and restoring UES resting pressure were also confirmed by manometry studies [13, 14]. These findings suggest that the observed improvements in swallowing and UES function were not only due to changes in mechanics aspects of cricopharyngeal sphincter, but also upstream neuroplastic changes. This hypothesis provides the rational for the current investigation on the influence of the modified balloon dilatation therapy on neural excitability in persons with dysphagia due to stroke.

Transcranial magnetic stimulation (TMS) is a non-invasive method to explore the treatment-induced neuroplastic changes. The amplitude of motor evoked potentials (MEPs) induced by TMS are used extensively and reliably to assess the effect of swallowing treatments on corticobulbar excitability [15–18]. Whereas MEPs which is measured in a resting state do not provide a direct measurement of a functional swallowing task, it is necessary to explore the linkage of corticomotor excitability and biomechanical characteristics of swallowing. Measure of swallowing timing, such as oral and pharyngeal transit time, swallowing response time, UES opening time, have been used to determine the association between swallowing functions with MEPs after various treatments (sensory stimulations, repetitive TMS, electrical stimulation) on swallowing [19–21]. These studies failed to report the corresponding changes in temporal measures of swallowing when MEPs amplitudes were modified [16, 22, 23]. The current paradigms have several notable limitations. One limitation of these studies is their approach for eliciting MEPs. Moreover, MEP changes were often recorded immediately (several minutes or hours) either after single treatment [24–26] or several days’ treatment (usually less than 1 week) [27]. Therefore, the long-term effects of these treatments on MEP fluctuations are unknown. Another limitation of prior work is its reliance on temporal measures of swallowing performance, which are known to be variable across participants [28, 29]. In contrast to temporal measure, kinematic measures of swallowing performance, such as UES opening diameter (UOD) and hyoid displacement (HD), have the advantage of being direct indicators of the mechanical effect of dilatation providing the specific quantitative change of the pharyngeal swallowing structures [30, 31]. Few researchers, however, have studied the correlation between MEP change and kinematic parameters. Therefore, it is imperative to couple MEP changes with structure displacements of modifications.

This study aimed to assess (1) whether modified balloon dilatation therapy could enhance human corticobulbar pathway excitability and (2) if these excitability changes were related to their displacement of hyoid and extent of UES opening in patients with brainstem stroke.

## Methods

### Subjects

Thirty patients with unilateral brainstem stroke were recruited from the Third Affiliated Hospital of Sun Yat-sen University and the Guangdong Second Provincial Traditional Chinese Medicine Hospital in China between October 2012 and November 2014. The diagnosis of brainstem stroke was determined by full clinical and neurological assessment and brain magnetic resonance imaging (MRI).

#### Inclusion Criteria

Patients were included based on the following criteria: age of 40–70 years, onset of stroke within 3–12 months and displaying sufficient level of cognition to receive interventions and evaluations by scoring at least 23 on minimental state examination [32]. Dysphagia was determined by a clinical swallowing evaluation and then further confirmed by videofluoroscopy swallowing study (VFSS). Both tests were rated by a single speech therapist who had at least 10 years of clinical experience in performing both assessments. For clinical screening, the examiner asked the patients to swallow a small volume of water (30 ml) and watched for signs of dysphagia (oral residue, coughing, choking, delayed swallowing, bolus holdup, throat clearing, reduced laryngeal/hyoid elevation, multiple swallows required, and nasal regurgitation). Exhibiting at least three symptoms and signs was enough for inclusion. The patients whose VFSS indicated that UES could not open, UES could not open completely, or UES opened at the wrong time, which resulted from lack of coordinated contraction of the pharyngeal muscle and UES, were eventually included. All patients were totally or partially dependent on tube feeding to meet their nutritional needs. All study participants had a nasopharyngeal tube present at the time of enrollment.

#### Exclusion Criteria

Patients with a previous history of neurological diseases, mental diseases, swallowing problems, reduced consciousness, epilepsy, metallic material (e.g., plates in the head or neck, or pacemakers), as well as patients who received drugs that might interfere with TMS (e.g., tranquilizers or antiepileptic drugs) were excluded. Patients were also excluded if they had a malignant disease, prior pharyngeal surgery, or radiotherapy, or suffered from headache, severe throat pain, or bleeding during the experiment.

#### Randomization and Blinding

Patients were randomly divided into dilatation and control groups by a computer-generated randomization sequence. One physician generated the random allocation sequence and enrolled participants, and then assigned participants to interventions. The therapist was aware of the allocation, but all the physicians who conducted evaluation procedures (including MEP measurement and VFSS data analysis) were blinded to group assignment. Informed consent was obtained from all participants. The study was approved by the local ethics committee of the Third Affiliated Hospital of Sun Yat-sen University (No. [2013]2-06).

### Treatment Protocol

#### Dilatation Group

Patients received modified balloon dilatation therapy combined with conventional therapies, and each of them was conducted once per day. The modified dilatation protocol has been detailed previously [11]. The nasopharyngeal feeding tube was removed in this group for placement of the Foley catheter per dilation protocol. After topical nasal anesthesia with 1% tetracaine hydrochloride solution, a double channel Foley urethral catheter (#14) with a deflated balloon (Well Lead Medical Co. Ltd., Guangzhou, China) was inserted through the nasal cavity into the mid-esophagus, and placement was confirmed by normal phonation. An assistant inflated the balloon with 3 ml water, and then another therapist gently pulled out the catheter until it was blocked. It was estimated that the balloon was supposed to be just under the lower margin of the UES [12]. A mark was made on the catheter near the nose. During dilation, the therapist tried to pull out the balloon through the UES, while the patient was instructed to swallow with effort until the balloon slipped out from the UES. Once the balloon crossed the UES, water was immediately drawn back into the injector. The procedure was repeated 5–8 times per session, 30 min each, for five consecutive days per week. The volume of water was increased incrementally by 0.5–1 ml daily (not more than 9 ml), depending on the degree of UES opening. Aerosol inhalation of budesonide suspension (1 mg) mixed with saline (5 ml) was performed immediately after each session to prevent mucosal edema. Slight throat pain can be rapidly relieved after inhalation. The patients in this group also received 30 min of the conventional therapies that in the control group received once daily, 5 days per week.

#### Control Group

Each patient assigned to this group received 30 min of conventional therapies, twice daily, 5 days per week. These therapies included: effortful swallow [33] (10 repetitions per day), Mendelssohn’s maneuver [34] (10 repetitions per day), supraglottic swallow [35] (10 repetition per day), and postural compensation of head rotation. Patients kept their nasal tube to compare with the Foley catheter in dilatation group during treatment.

All participants received the treatments at the time of hospitalization. The study was terminated when any of the followings occurred: (1) treatment had been administered for 3 weeks, (2) patients were completely dependent on oral feeding, and no longer were dependent on the supplemental tube feeding.

### Data Collection

A submental muscle response variable from independent control and dilatation subjects was recorded. Baseline MEP and VFSS were taken on the day before the first treatment and measurements were repeated on the day after the last session. The NIHSS score and FOIS were also determined.

#### MEP Recording

Patients were seated comfortably in a chair without movement during assessment, with slight extension of the head. The experimenter would clean the areas under the chin and overlying the ramus of the mandible with an alcohol swab, shaved any beard and then rubbed the skin of the patient with a body scrubbing cream if necessary to ensure that impedance of submental skin was lower than 5 kΩ (measured by the electromyogram). MEPs were recorded from bilateral submental muscle group (mostly mylohyoid). A pair of shielded bipolar silver chloride surface electrodes were used as recording electrodes. The active electrode was positioned 2 cm lateral to the mid-point of the chin and hyoid bone. The reference electrode was mounted over the hyoid, about 2 cm medial to the active electrode. A ground electrode was placed over either arm. All electrodes were connected to a portable electromyography (EMG) and evoked potential system (NTS-2000, NCC Medical Co. Ltd., Shanghai, China) that filtered (bandpass set at 0.02–10 kHz), rectified, and amplified the EMG signal (sweep rate of 5 ms/div, gain of 0.2 mv/div) with a sampling rate of 200 kHz. Correct placement was verified in the EMG monitor by asking the patients to perform a tongue press against the hard palate, showing corresponding activities. Participants had to avoid swallowing and relax without any body motion during stimulation.

**Fig. 1:**
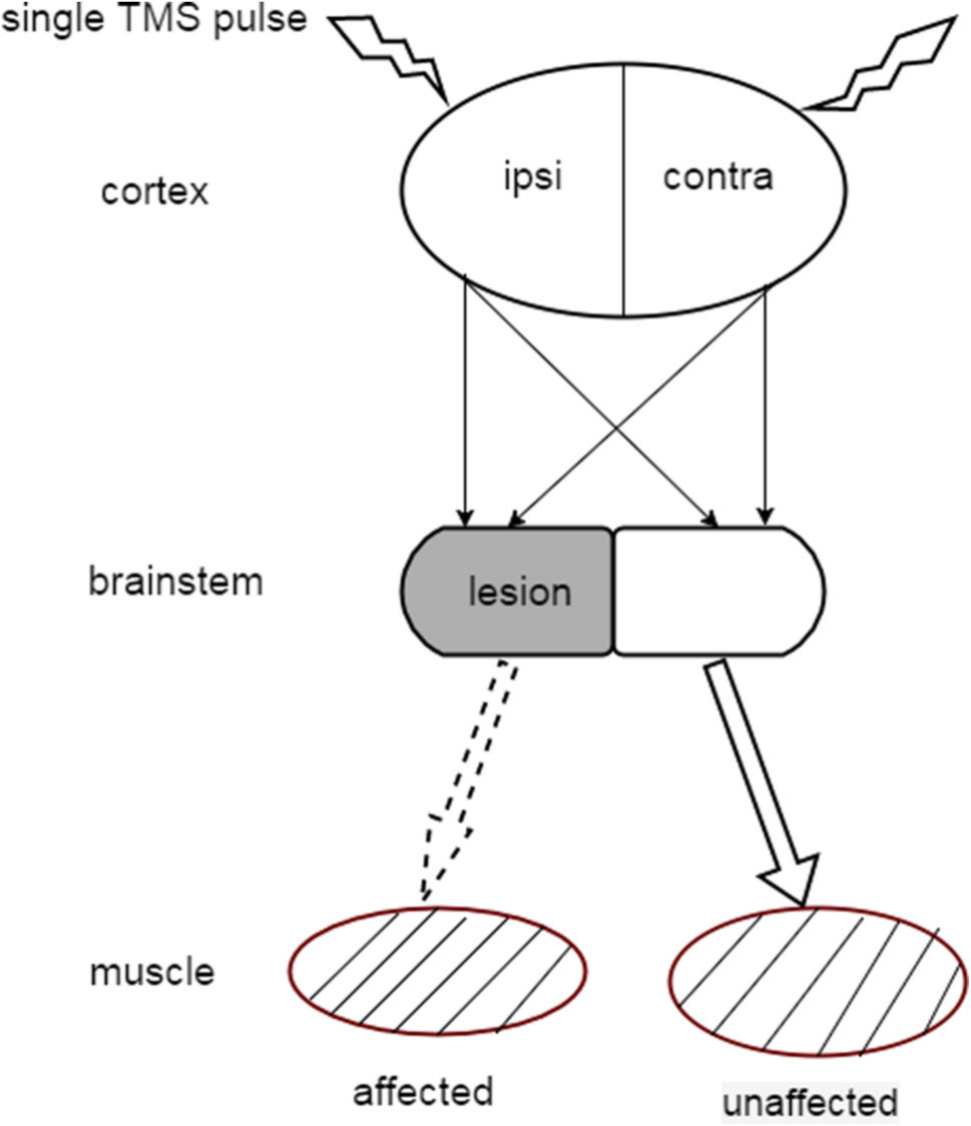
Schematic diagram of submental MEP. The cortical stimulation ipsilateral to the brainstem lesion would be classed as the ipsilateral. The muscle ipsilateral to the brainstem lesion was regarded as “affected muscle” due to the nerve supply impairment*. Affected* the affected submental muscle, *unaffected* the unaffected submental muscle, *ipsi* the cortex ipsilateral to the brainstem lesion, *contra* the cortex contralateral to the brainstem lesion. The *dashed arrow* represents the affected projection

MEPs were recorded twice for each patient at the baseline and the endpoint of all interventions. Due to the bilateral innervation of submental muscle, single cortical stimulation of either right or left hemisphere always evoked MEPs on bilateral submental muscle in a normal condition (see in Fig. 1). For each subject, there will be four kinds of MEPs: the affected muscle MEP induced by ipsilateral cortical stimulation (IA), the unaffected muscle MEP induced by contralateral stimulation (CU), the affected muscle MEP induced by contralateral stimulation (CA) and the unaffected muscle MEP induced by ipsilateral stimulation (IU).

#### Cortical Stimulation

Single-pulse TMS was performed using a hand-held figure-of-eight coil (90 mm outer diameter) connected to a magnetic stimulator (CCY-II, YIRUIDE Medical Equipment Co. Ltd., Wuhan, China) with maximal output of 2.2 T. The stimulation procedures for all participants were conducted by the same trained physician.

Each patient wore an elasticated head cap. The experimenter marked the nasion–inion and interaural lines, and then located the vertex (*C*_*z*_) at the intersection of the above two lines according to the international 10–20 system. A grid of 1 cm squares covering a 10 × 10 cm area was then drawn on each side of the vertex by marking the scalp with an indelible marker. The anteroposterior rows of this grid were aligned parallel to the sagittal plane, while the mediolateral rows were aligned 90° to the sagittal plane.

TMS was then applied separately on random hemisphere in an anteroposterior direction, with the plane of the coil parallel to the scalp surface and the handle of the coil approximately 45° to the mid-sagittal line. The optimal sites for evoking the maximum submental MEPs from both hemispheres were identified as hotspots. According to Hamdy et al. [36] and Plowman-Prine et al. [37], a hot spot was positioned 2–4 cm anteriorly and 4–6 cm laterally.

We moved the coil one grid at a time in this area at an intensity of 100% of the stimulator output to obtain a response. Once identified, the location axis of the hotspots related to *C*_*z*_ was recorded and marked as “x” on the cap to ensure that the site could be reproducibly obtained. A series of cortical stimuli over this position was then performed, gradually decreasing by 5% stimulator output steps until threshold intensity was found that evoked pharyngeal EMG responses of 50 μV in at least 5 of 10 consecutive trials. Ten repeated single-pulse stimuli from both hemispheres were then applied at an intensity of 110% threshold with an interval of 5 s. Cortical stimulation was always performed between swallows.

#### Kinematic Measurements

Kinematic variables were rated from VFSS which was conducted before enrollment and after treatments. VFSS was conducted using a gastrointestinal X-ray machine (Toshiba DBA-300, Toshiba Co. Ltd., Tokyo, Japan) in a routine protocol [38].The only difference was that a metal sphere with a diameter of 8 mm was tapped as reference for further kinematic analysis. The videos were recorded using a VFSS digital acquisition unit (Longest Ltd., Inc., Guangzhou, China) at 30 frames/s.

### Data Analysis

The amplitudes of the cortically evoked EMG responses across 10 stimuli were combined to produce a mean value for each individual. The amplitude was defined as maximum peak-to-peak voltage of the EMG response expressed in mV. For absent EMG responses amplitudes were noted as 0 mV.

Kinematic parameters were measured based on frame-by-frame analysis of VFSS videos by VitualDub (GNU General Public License) and Image J2x (National Institute of Mental Health, Bethesda, MD, USA). The image preprocessing and measurement procedure was previously described [19, 38–40]. The details of formula for measurements were included in Appendix. HD was defined as the maximal displacement of hyoid excursion calculated by the maximal anterior and superior displacement during 5 ml barium swallows (see Appendix Fig. a, b). UOD was defined as the maximal anteroposterior diameter of UES opening, which was the widest portion of the bolus flow at the UES level during 5 ml liquid barium swallows (see in Appendix Fig. c). We only included the UES data of swallowing thick liquid food, because they were considered as a safer and most common type of food for patients with brainstem stroke in clinical practices [41].

### Statistical Analysis

Normally distribution of data was determined by the Shapiro–Wilk test. Homogeneity of variance was measured with Levene’s test. The Mann–Whitney *U* test was used to compare NIHSS and FOIS scores between two groups. The *χ* ^2^ test was used to compare the categorical data of baseline characteristics. A three-way repeated ANOVA analysis was used to test the group (dilation vs. control), time (pre vs. post), and MEP laterality (IA, IU, CA, and CU) effect on MEP amplitudes. Mauchley’s test was used to test for sphericity and if violated tests with adjusted df were used (Greenhouse–Geisser). Turkey HD test was used to post hoc comparisons. We used the paired comparisons of pretreatment MEP amplitudes between the affected and unaffected muscle within subject to validate the MEP results. Spearman’s correlations were used to test the relationship of the amplitude change of MEP and UOD as well as HD. *p* < 0.05 was considered to be statistically significant. Effect size was estimated in partial eta-squared ($\eta_{\text{p}}^{2}$). All statistical analyses and graphs were conducted using SPSS 21.

## Results

### Patient Demographics

Thirty-nine patients were recruited, but nine of them were excluded because of previous neurologic diseases. Demographics of all patients are shown in Table 1. Although eight of them had cortical and subcortical lacunars infarction besides brainstem lesions, they had no sign of limb weakness, spasticity, tongue atrophy, or tremor, and their NIHSS scores were not more than 5. There were no differences in time from onset (*Z* = 0.766, *p* = 0.381) and age (*Z* = 0.190, *p* = 0.803) between groups. All patients received therapies for more than 2 weeks, and 80% of them received treatments for 3 weeks.

**Table 1:** Patient demographics

Baseline characteristics	Dilation *N* = 15	Control *N* = 15	*p*
Age (mean ± SD, years)	57.7 ± 8.8	57.9 ± 9.3	0.80
Gender (F/M)	4/11	5/10	0.69
Time from onset (mean ± SD, months)	4.3 ± 2.6	4.5 ± 2.3	0.57
NIHSS	3	2	0.64
Laterality of stroke (L%)	66.7	60	0.71
Lesion level			
Medulla (%)	86.7^a^	60	0.09
Mixed with subcortical lesion^b^ (%)	33.3	20	0.41
Occurence of MEP			
Affected (%)	60	66.7	0.71
Unaffected (%)	80	80	0.67
Treatment sessions (mean ± SD)	14 ± 1.8	14.4 ± 1.4	0.59
Rate of aspiration pneumonia (%)	33.3	53.3	0.27

^a^Including two patients who combined with pontine lesion

^b^Minor lesions levels including basal ganglia, thalamus, focal coronal radiation, focal frontal cortex

### Functional Outcomes (FOIS Score and Tube Dependence)

All patients were totally or partially dependent on tube feeding when participating in this study. After treatment, only two patients in dilatation group and nine in control were still fed by tube (control vs. dilatation: *Z* = 7.03, *p* = 0.008). At baseline, the dilatation and control groups’ FOIS scores were not different (*Z* = −1.72, *p* = 0.860). The treatment effect was greater for the dilation group than the control group (*Z* = −2.34, *p* = 0.019). In the dilatation group, FOIS scores improved from a median of 1 to 4 after treatment (*Z* = 3.47, *p* < 0.001). Median FOIS scores only improved from 1 to 3 in the control group (*Z* = 3.25, *p* = 0.01).

### MEP Results

All patients were tolerant of TMS. The hot spots of stimulation on the hemispheres were located at 2–5 cm laterally and 3–6 cm anteriorly to the vertex for the right and 2–5 cm laterally and 3–7 cm anteriorly to the vertex for the left.

#### The Occurrence of MEP Responses

Cortical stimulation did not evoke MEPs in all participants. Before treatment, bilateral cortical pulse did not elicit a discernible response of the affected muscle in six of participants in the dilatation group and five in the control group. Baseline MEPs of the unaffected muscle were also not detected in three patients of each group. After treatment, MEPs induced by affected muscle were still absent in two patients in dilatation group and four in control group. Treatment did not affect the occurrence of the MEPs in unaffected muscles.

#### Amplitudes Comparisons

**Fig. 2:**
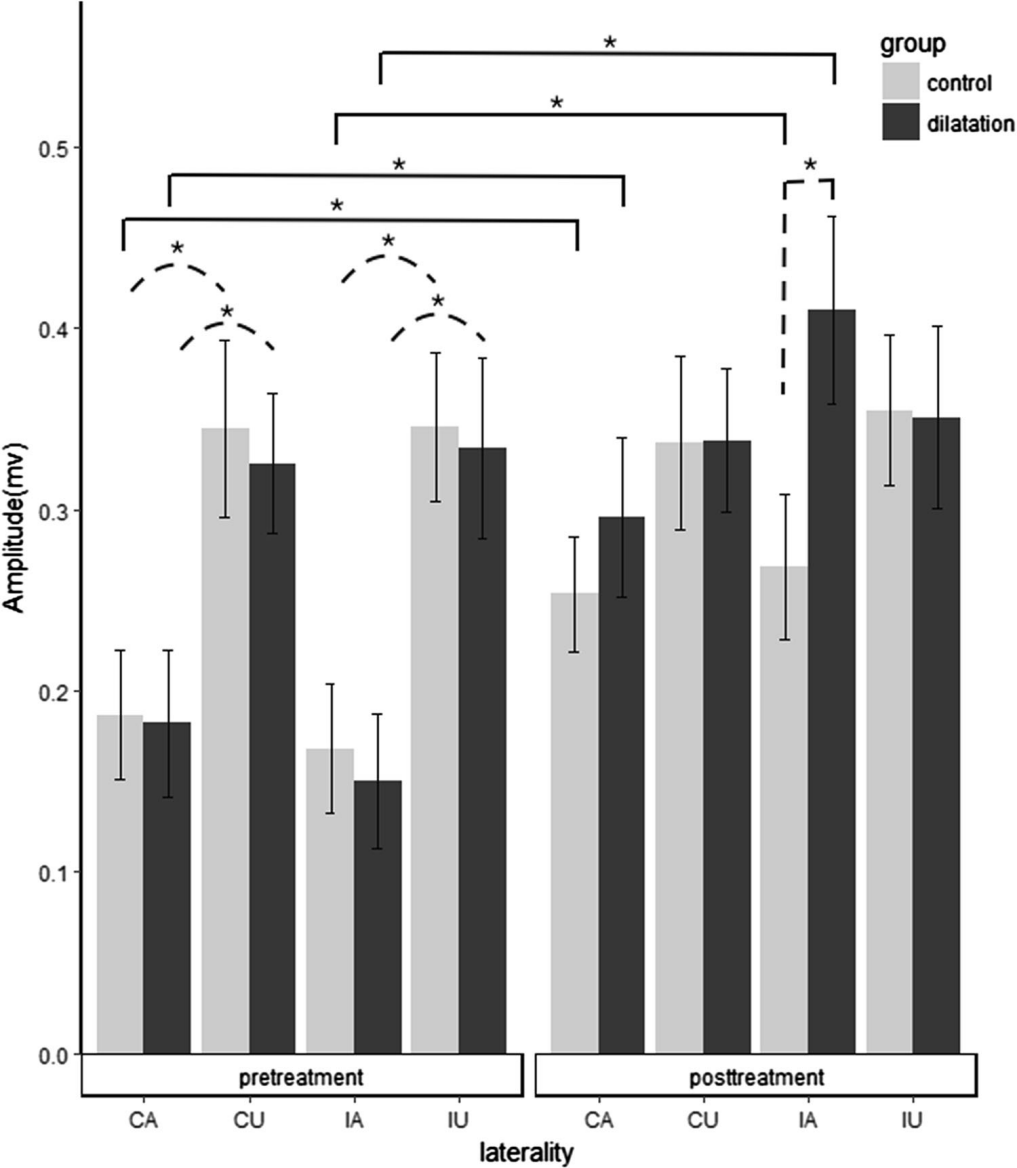
Comparisons of the amplitudes of submental MEP within group and between groups. *Dashed bracket* represents the comparison between two groups for each laterality. *Dashed parenthesis* represents the comparison of the affected and unaffected submental pretreatment MEP within group. *Brackets* represent the comparisons of pre- and post-treatment MEPs. *p < 0.05, *error bar* represents standard error. *IA* the affected submental MEP induced by ipsilateral stimulation, *CA* the affected submental MEP induced by contralateral stimulation, *IU* the unaffected submental MEP induced by ipsilateral stimulation, *CU* the unaffected submental MEP induced by contralateral stimulation

There were main effects on time, group, and MEP laterality (*F* = 3.460, *p* = 0.02, $\eta_{\text{p}}^{2}$ = 0.08). The amplitudes of submental MEPs changed over time (*p* < 0.001, $\eta_{\text{p}}^{2}$ = 0.34). Both the interactions of group * time (*p* = 0.002, $\eta_{\text{p}}^{2}$ = 0.08) and laterality * time (*p* < 0.001, $\eta_{\text{p}}^{2}$ = 0.34) had significant effect. All the post hoc comparisons are showed in Fig. 2.

Before treatment, the amplitudes of MEP between groups for each laterality were compared. No significant differences of amplitudes were found in four lateralities (IA: *p* = 0.76, CA: *p* = 0.94, IU: *p* = 0.85, CU: *p* = 0.74). Following comparisons within group demonstrated that the MEP amplitudes of the affected muscle were significantly smaller than the unaffected in each subject in both groups (dilation IA vs. IU: *p* = 0.001, dilation CA vs. CU: *p* = 0.041, control IA vs. IU: *p* = 0.007, and control CA vs. CU: *p* < 0.001, respectively).

After treatment, only the amplitudes of the affected submental MEP evoked by ipsilateral cortical stimulation (IA) were significantly larger in the dilation group than in the control (*p* = 0.02, *d* = 0.79). No between group differences were found in the comparisons of unaffected MEP (IU and CU) and the affected MEP evoked by contralateral stimulation (CA).

Pairwise comparisons of pre- and post-treatment amplitudes showed that the amplitudes of the affected MEPs induced by bilateral cortical stimulation increased following treatment in both the dilation group (IA: *p* < 0.001, *d* = 1.50, CA: *p* < 0.001, *d* = 0.69) and the control group (IA: *p* < 0.001, *d* = 0.68, CA: *p* = 0.01, *d* = 0.51). No treatment effects on amplitudes of IU and CU were found in the dilatation group (IU: *p* = 0.53, *d* = 0.09, CU: *p* = 0.63, *d* = 0.08), as well as control group (IU: *p* = 0.73, *d* = 0.06, CU: *p* = 0.76, *d* = 0.04).

### VFSS Parameters

**Fig. 3:**
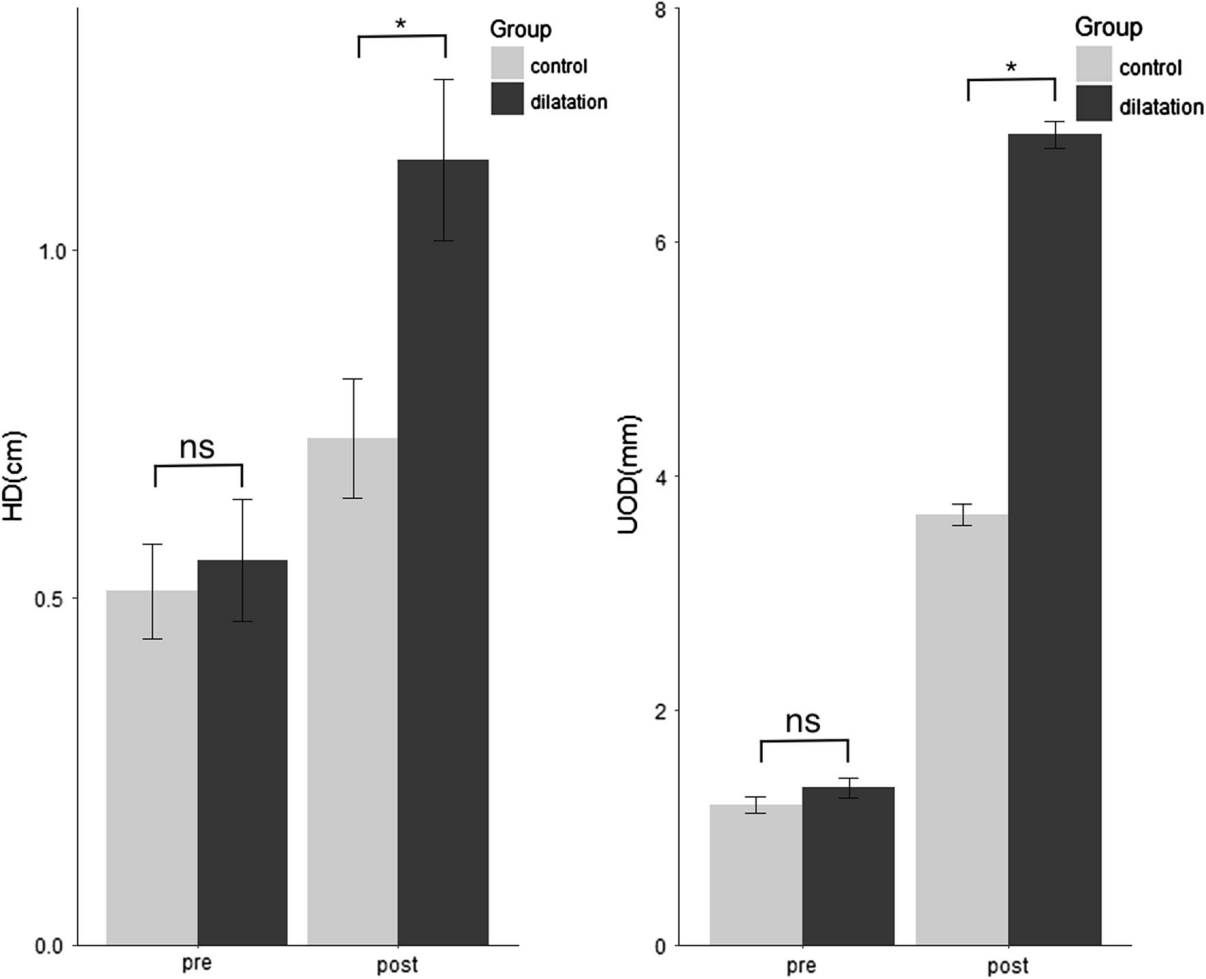
Comparisons of hyoid displacement and the diameters of UES opening between two groups. *p < 0.05, *NS* represents no significance, *error bar* represents standard error. *HD* hyoid displacement, *UOD* maximal diameters of UES opening

No significant differences were detected for HD as well as UOD before treatment (*p* = 0.78 and 0.69, respectively). Otherwise, after treatment, HD and UOD significantly increased in the dilatation group as compared to those in the control group (*p* < 0.001 for HD, and *p* = 0.03 for UOD, respectively), as indicated in Fig. 3.

### Correlation of Changes in Amplitudes of MEPs and VFSS Parameters

**Fig. 4:**
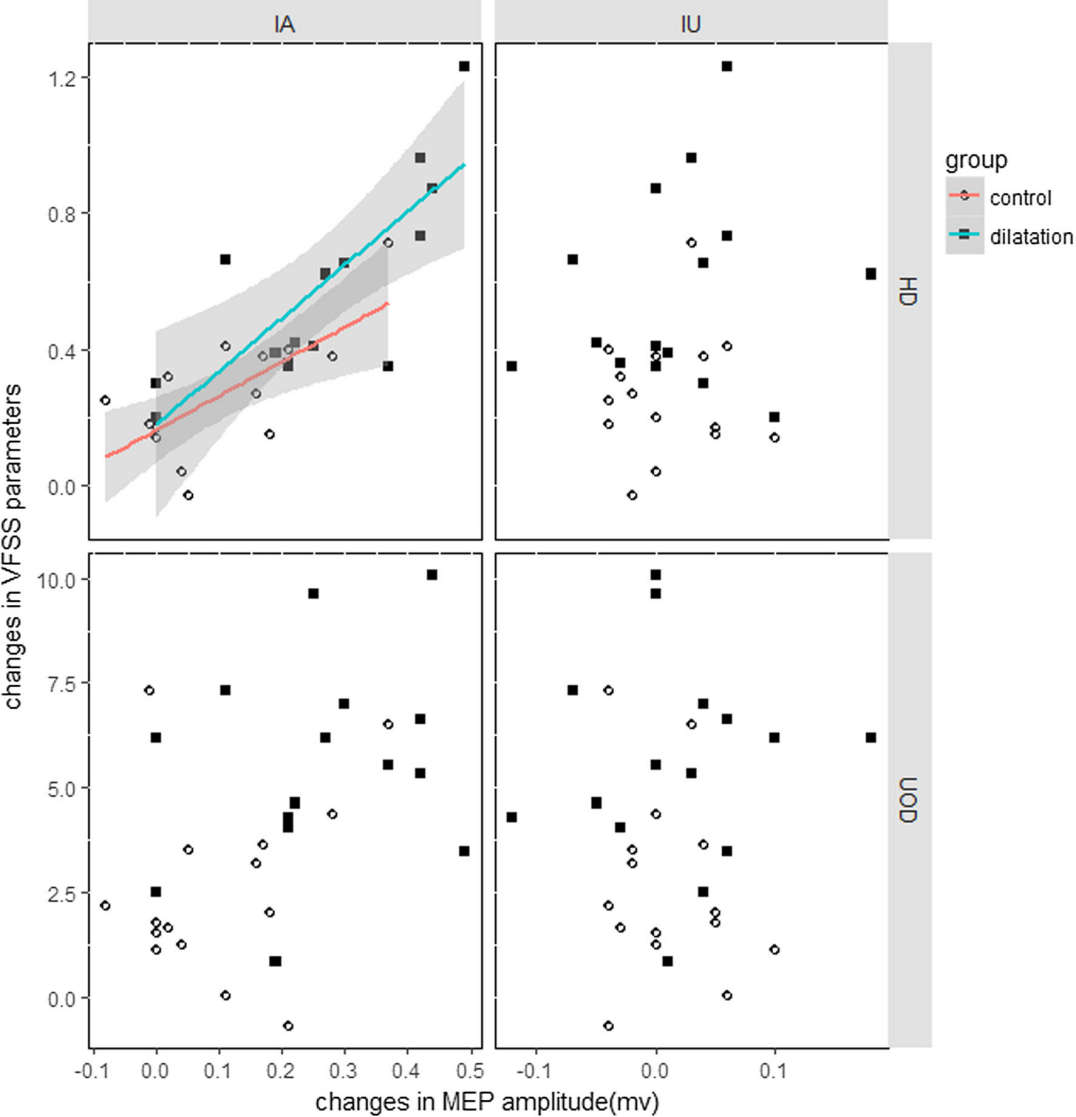
Correlation of improvement of kinematic parameters and amplitude changes of submental MEP evoked by ipsilateral stimulation before and after treatment in both groups. Significant linear fit lines were showed between the affected MEP and HD in two groups

We only examined the correlation between the affected and unaffected MEP evoked by ipsilateral cortical stimulation to VFSS parameters (see Fig. 4). There were positive linear correlations between the amplitude change of affected submental MEPs, and the HD in both the dilatation (*R* ^2^ = 0.51, *p* = 0.03) and control groups (*R* ^2^ = 0.39, *p* = 0.01); in contrast, there was no correlation between the maximum extent of UES opening and MEP amplitude of bilateral submental muscle in the dilatation groups (affected: *p* = 0.29, unaffected: *p* = 0.87) and the control group (affected: *p* = 0.33; unaffected: *p* = 0.49, respectively).

## Discussion

The primary goal of this study was to determine impact of the novel modified dilatation intervention on corticobulbar excitability, based on the bilateral submental MEPs evoked by bilateral hemisphere stimulation. To the best of our knowledge, this is the first study to investigate the MEP changes after as long as 3 weeks of UES dilation intervention in patients with brainstem stroke. Following the intervention, increased MEP amplitudes were obtained for submental muscles that were ipsilateral to the lesion, and correlated to the improvement of HD. This finding suggests that during the recovery of the ipsilateral bulbar pathway may be the main cause of the pharyngeal function improvement.

### Reliability and Validity of MEP Measurements

Several design features were implemented to maximize the reliability and validity of the responses, because MEPs are known to be sensitive to variations in stimulation location, stimulation intensity and the state of the target muscle contraction. In order to ensure pre- and post-treatment consistency of stimulus location, we used a grid of 1 × 1 cm to identify hot spots through the experiment. Stimulus intensity was maintained as 110% threshold, although it may have different thresholds between hemispheres. Furthermore, all the MEP tests were conducted in the resting state without any oropharyngeal and facial muscle activities in order to avoid the effect of different levels of cortical facilitation resulting from muscle contraction.

To validate present study, we compared the MEP amplitude of the affected and unaffected muscle when imposed the stimulus on the same hot spot. Since brainstem lesions cause impairment in the connection between periphery muscle and the corresponding cortex, it is reasonable that the amplitude of affected MEP was decreased or even absent, compared to the unaffected before treatment. As expected, the amplitude of affected submental MEPs was indeed lower than that in the unaffected muscle in both groups. Notwithstanding, the amplitudes of MEPs (~350uv) of the unaffected muscle, which should be normal in our subjects were lower than the normal values previously reported by Gallas et al. (400–600uv) [42]. A possible explanation was that serial stimulus had been performed prior to the stimulation evoking the included MEP response when we tried to figure out the hot spots. It might result in similar efficacy of repetitive interventions at frequency of lower than 1 Hz which was considered to inhibit cortical excitability.

### Speculation About Neurologic Mechanism of Dilatation Therapy

In this study, the observed increase in MEP amplitudes following treatment in both groups indicated that both dilatation and conventional therapy enhanced the corticobulbar excitability, although the response of the dilatation group was significantly larger than those of the control group. Several investigators have previously suggested that the conventional therapies, such as effortful swallow and Mendelssohn maneuver, enhance cortical activities [43, 44]. Our prior fMRI study confirmed this suggestion, but further demonstrated that, in comparison to conventional therapy, dilatation therapy engaged more cortical regions [45]. We speculated that the effects of the dilatation were limited to the ipsilateral bulbar pathway, because comparisons between groups showed that only the amplitude of affected submental MEPs induced by ipsilateral hemispheric stimulation significantly increased, while that of unaffected submental MEP was not different.

All descending cortical inputs in association with periphery sensory feedback are integrated by the brainstem which contains two swallowing hemi-CPGs [46]. After unilateral brainstem stroke, the synchronization of hemi-CPGs as well as ipsilateral oropharyngeal swallowing muscle can be impaired. When the balloon expanded the UES, it may produce tactile and pressure stimulation of the UES, while the pharyngeal nerve plexus could be stimulated, inducing a swallowing reflex. It is known that the oscillations of CPG can be entrained by sensory neurons [47]. In addition to sensory stimulation, the modified dilatation therapy makes it possible for severe dysphagic patients to be treated in a graded manner in a swallowing balloon task without risks of aspiration. This graded swallowing task might strengthen the drive of the swallowing cortex. Studies have demonstrated that a voluntary swallowing with task-oriented biofeedback could involve more brain activation [48] and rehabilitate swallowing function [49]. When we changed the balloon volume, a real-time target extent of UES opening can easily be provided as a feedback for patients. Dou et al. [12] also demonstrated that this active dilation therapy can provide greater improvement of oral feeding than passive dilation. Although few studies focused on the changes of cortical excitability after brainstem stroke, cortical compensation in patients with brainstem injury such as X-linked bulbospinal neuronopathy was observed by Dziewas et al. [50]. We speculated that repeated dilatation therapy might restore the synchronization of hemi-CPGs through increased cortical input and sensory stimulation. Certainly, these should be confirmed by further direct brain imaging evidence or other electrophysiological methods, such as electroencephalogram, which could explore the complex neural network regulating the act of swallowing.

### Evidence from Kinematic and Functional Outcomes

Decreased hyoid anterior and superior excursion and impairment of UES relaxation usually coexist in patients with brainstem stroke. Our findings showed that modified dilatation therapy could improve UES opening as well as hyoid excursion. These kinematic changes were also accompanied by a significant improvement of oral feeding level. It indicated that this treatment not only dilated the cricopharyngeal muscle, but also motivate the pharyngeal muscles, as demonstrated by a previous study [14].

Correlation analysis further demonstrated that the alteration of affected submental MEP evoked by ipsilateral cortical stimulation was positively correlated with the improvement of hyoid movement in both groups. Although the causal connection could not be addressed, the data still supported the hypothesis that the excitability of the affected descending motor projection had functional correlate during dysphagia recovery in chronic brainstem stroke. It is worth mentioning that the correlation between UOD with submental MEP was not found. This may be because the recording site of submental MEP was mainly at the location of mylohyoid muscle which raises and stabilizes the hyoid. Although mylohyoid muscle is not the target muscle directly imposed by dilatation therapy, it is the largest and most important muscle in the suprahyoid group that can be easily recorded by surface electrodes. Moreover, the suprahyoid muscles is a key component in the pharyngeal phase of swallowing that provides the major distracting forces to overcome the tone-generating muscles in the UES [51]. In contrast, a direct MEP of UES is not available except when it is measured using invasive needle electrode [52]. On the other hand, UES opening during swallowing is determined by several factors. The extent of opening not only depends on the relaxation of UES tone-generating muscle which is related to nerve excitability, but also the bolus volume [53], traction of anterior and hyoid–laryngeal complex, and pharyngeal propulsion [54].Therefore, the diameter of UES opening may not vary with amplitude of submental MEP, although they both improved more in the dilatation group than in the control.

Given the nature of this treatment, it was difficult to keep the patients and therapists blinded, and this posed a limitation. The absence of sham dilation for the control group may be another limitation to reveal the underlying neural mechanisms; however, patients in control group carried with a nasopharyngeal tube during treatment sessions, which can be an alternative control for possible influence of catheter itself during swallowing.

## Conclusion

Modified catheter balloon dilatation therapy may increase the excitability of the affected corticomotor projections in patients with unilateral brainstem stroke, correlated with the improvement of HD. This should be verified by brain imaging in the future.

## Electronic supplementary material

Below is the link to the electronic supplementary material.

Supplementary material 1 (TIFF 4678 kb)

Measurements of kinematic parameters in VFSS. a The resting position of hyoid which coordinated by (X 1, Y 1), C 4 represents the fourth vertebral coordinated by (C 4 X 1, C 4 Y 1), b the maximal hyoid elevation during pharyngeal swallowing, coordinated by (X 2, Y 2), C 4 represents the fourth vertebral coordinated by (C 4 X 2, C 4 Y 2), hyoid anterior displacement: HA = (X 2 – X 1) – (C 4 X 2 − C 4 X 1), hyoid superior displacement: HS = (Y 2 – Y 1) – (C 4 Y 2 − C 4 Y 1), hyoid displacement maximum: $${\text{HD}} = \sqrt {{\text{HA}}^{2} + {\text{HS}}^{2} }$$$\text{HD}=\sqrt{{\text{HA}}^{2}+{\text{HS}}^{2}}$$${\text{HD}} = \sqrt {{\text{HA}}^{2} + {\text{HS}}^{2} }$$$\text{HD}=\sqrt{{\text{HA}}^{2}+{\text{HS}}^{2}}$ and c UES opening diameter: maximally opened diameter during bolus transit across the sphincter within the pharyngoesophageal junction (about C 4–C 6 level). UES represents upper esophageal sphincter.
