# Supplementary figures and images for: Change in Excitability of Cortical Projection After Modified Catheter Balloon Dilatation Therapy in Brainstem Stroke Patients with Dysphagia: A Prospective Controlled Study
Source: Dysphagia. 2017 May 26;32(5):645–56. doi: 10.1007/s00455-017-9810-6 (PMC5608794; doi:10.1007/s00455-017-9810-6)

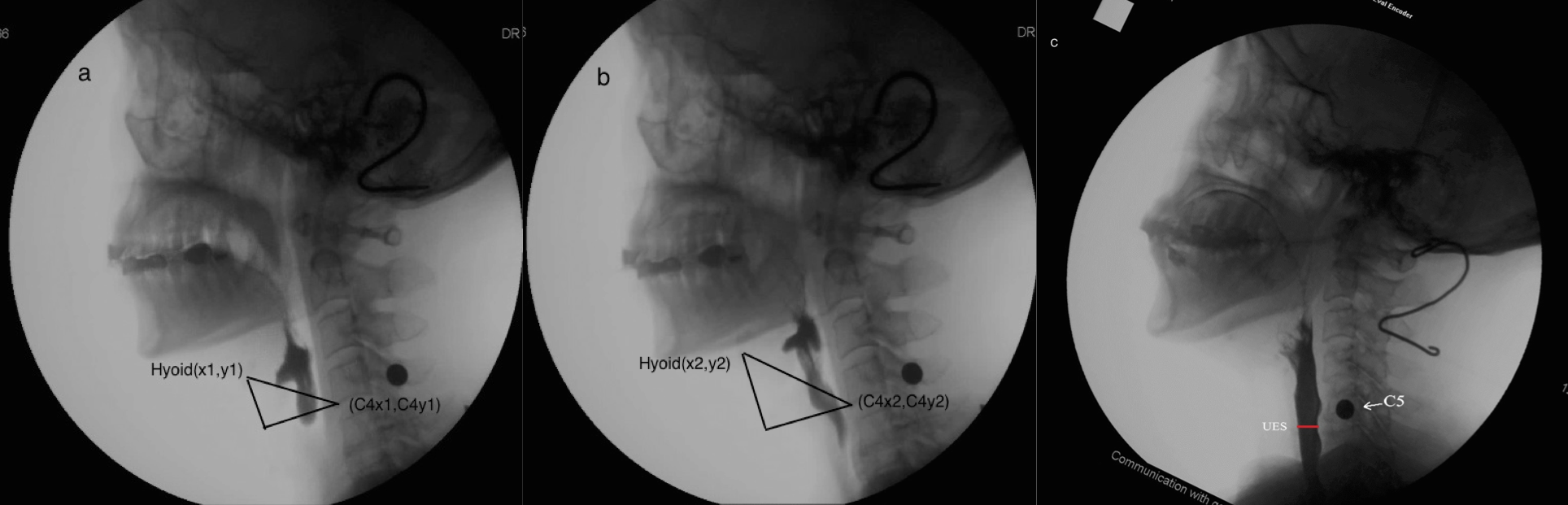

Supplement: Supplementary file 1 — Supplementary material 1 (TIFF 4678 kb) [file 455_2017_9810_MOESM1_ESM.tiff]
